# Longitudinal associations between family functioning and generalized anxiety among adolescents: the mediating role of self-identity and cognitive flexibility

**DOI:** 10.1186/s40359-024-01908-1

**Published:** 2024-07-18

**Authors:** Ran Ma, Qian Zhang, Chunyang Zhang, Wei Xu

**Affiliations:** 1 Beijing Key Laboratory of Applied Experimental Psychology, Faculty of Psychology, National Demonstration Center for Experimental Psychology Education, Beijing Normal University, Beijing, China; 2 School of Psychology, University of Leeds, Leeds, UK; 3 China Academy of Civil Aviation Science and Technology, Beijing, China

**Keywords:** Generalized anxiety, Family functioning, Self-identity, Cognitive flexibility, Two-wave study

## Abstract

**Background:**

Generalized anxiety (GA) is showing a high prevalence among adolescents nowadays; investigations on influencing factors and potential mechanisms are important to inform intervention development. The present two-wave study investigated the ways in which family functioning predicted GA six months later among adolescents, by considering the mediating role of self-identity and cognitive flexibility.

**Methods:**

Adolescents were recruited from 27 randomly selected classes in two secondary schools in Chongqing and Fujian Province, China. Survey questionnaires assessing family functioning, self-identity, cognitive flexibility and GA were obtained from 1223 adolescents (*M*_*age*_ = 13.14, *SD* = 1.35) at two time points of the 6-month interval.

**Results:**

The association between family functioning (T1) and GA (T2) was significant (*r*= -0.152, *p* < 0.01). Self-identity and cognitive flexibility sequentially mediated the relationship between family functioning (T1) and GA (T2) (with the indirect effect = -0.005, 95% CI = -0.007~ -0.002) after controlling for age, gender, and GA at baseline. Cognitive flexibility also showed a significant and direct mediating effect (with the indirect effect = -0.008, 95% CI = -0.012 ~ -0.005).

**Conclusion:**

Findings indicated that family functioning can be a protective factor of GA, and self-identity and cognitive flexibility act as a crucial role in the association between family functioning and GA. Future studies should adopt more time points and long-term follow-up assessments using more robust approaches to improve the reliability of the study findings. Findings may offer some implications that building a harmonious, open and warm family and guiding adolescents to develop self-identity as well as more flexible cognitive style could be helpful to prevent and cope with anxious emotion.

## Introduction

Nowadays, adolescents are increasingly confronted with stressful situations and challenges in a highly competitive environment, leading to a high prevalence of anxiety problems [1, 2]. Generalized anxiety (GA) is one of the most common anxious emotions among adolescents. According to the DSM-5 [3], Generalized anxiety disorder (GAD) is characterized by exaggerated and uncontrollable worries about numerous daily routines with three or more somatic and cognitive symptoms. As for adolescents, they tend to excessively worry about their performance in school or social activities and feel less sense of control of worry [4]. The average prevalence rate of GAD is between 2.2% and 3.6% in children and adolescents [5]. According to the National Comorbidity Survey–Adolescent Supplement (NCS-A) conducted in 2010, the prevalence of GAD among community adolescents was nearly 2.2% [6], while another survey adopting an Australian nationally representative sample showed 2.3% [7]. There’s a 6-wave community study indicated increasing prevalence as aging and peaked at 4.51% at age 14 [8]. Evidence to date has established the relatively high rates of comorbidity in GAD in young people with other mental disorders, such as other anxiety disorders, and major depressive disorder [9–13].

It should be noted that there could be even more adolescents experiencing GA symptoms without being diagnosed with GAD [14, 15]. Data from several studies suggested that young adults suffering from GAD typically first developed related symptoms in early to middle adolescence and persist into adulthood [9, 16]. Early GA symptoms without appropriate interventions may increase the vulnerability of adolescents to other mental disorders, which can partially explain the high comorbidity rates of GAD [10]. It is therefore very important to investigate the influencing factors of GA in adolescents which can inform the development of preventive and treatment interventions. Since previous research has explored factors of GA from different aspects [17–19], a comprehensive explanation needs will be me specific factors may shed more light on the mechanisms of GA and offer more targeted implications.

### Family functioning and generalized anxiety

According to the Contrast Avoidance Model [20], when in an inhospitable family environment, adolescents face too much uncertainty about whether their needs will be met and emotional swings, and they may undergo continuous intra personal negativity as a means of preparing for unexpected parental behavior [21]. A growing body of research highlights the role of family relationships and interactions as key contextual factors in understanding GA in adolescents. For example, a 10-year longitudinal family study with a large sample of adolescents and young adults found that dysfunctional family functioning predicted a higher incidence of GAD [9]. According to the Circumplex model put forward by Olson et al. [22], the important dimensions of family functioning are family cohesion, which focuses on the emotional connection between family members, and flexibility, which refers to the way a family balances stability and change. Perceived maladaptive family functioning components such as low family/parent support and high family/interparental conflict have been connected with GA in adolescents [9, 23–25]. Dysfunctional family functioning was also shown to contribute to the occurrence of anxiety in youth [26, 27]. A systematic review conducted by Yap et al. [28] revealed that parental factors including less warmth, more inter-parental conflict, over-involvement, and aversiveness, which represent poor family functioning may act as risk factors for anxiety in adolescents.

It should be noted that the evidence cited above has been established in predominately white European or North American samples. Family factors are to an extent culturally determined [29–31]. A cross-cultural comparison involving adolescents from Western and Eastern cultures demonstrated cultural differences in the predictive power of family factors in adolescent anxiety [30]. To be specific, adolescents from Eastern cultures like Korea tend to show lower ratings of healthy family functioning and higher levels of anxiety, comparing with those from Western cultures [31]. In a study involving participants from North America and China, it was found that parents in Chinese families provide less autonomous support, leading adolescents to pursue external life goals and experience less well-being as a result [32]. The present study, therefore, aimed to extend the existing literature by examining the negative predictive association between healthy family functioning and GA in Chinese adolescents. In addition, the mechanisms mediating the relationship between family factors and adolescent anxiety are currently not well-understood. This study investigated the potential mediating role of two psychological factors, self-identity, and cognitive flexibility, in the association between family functioning and GA.

### The role of self-identity

Contrast Avoidance Model [21] also raised that, when individuals have no clear self-cognition or have a rather negative self-concept, out of “consistency motivation” or the need to maintain cognitive predictability, individuals tend to strongly reject positive feedback that is inconsistent with their negative self-concept in interpersonal relationships, and thus are more likely to experience negative emotions. According to Erikson’s theory of psychosocial development stages, it is the most prominent developmental task in adolescence to form a coherent and organized sense of identity [33]. The identity formation processes can be stressful and involve ongoing uncertainty and confusion about one’s self, and may therefore induce internalizing symptoms including GA [34]. There has been evidence showing that identity problems such as the process of unstable identity (i.e., *reconsideration of commitment*) [35] or the lack of a clear sense of self were associated with higher GA in adolescents [34–37].

Family can play an essential role in one’s identity formation. Evidence from cross-sectional and longitudinal studies has revealed an association between healthier family functioning and more adaptive identity development. For example, a 36-month longitudinal study found that the changes in adolescents’ perception of family functioning were strongly related to the variability in identity confusion [38]. Another five-wave longitudinal study indicated the protective effects of maternal support on adolescent identity development [39]. Dysfunctional family functioning has been frequently associated with childhood adverse experiences [40–43], which according to the Identity Disruption Model, could lead to disrupted identity and in turn result in psychological distress in later life [44]. Based on these theoretical and empirical findings indicating the associations between family functioning, self-identity, and GA, we proposed that self-identity may have a mediating effect on the relationship between family functioning and anxiety in Chinese adolescents.

### The role of cognitive flexibility

According to the Avoidance Model of Worry and GAD (AMW) [45, 46], in the process of trying to solve the problem, in order to eliminate the perceived threat and avoid the negative emotional experience that naturally occurs in the process of fear confrontation, there will be worry, a relatively ineffective cognitive attempt, thus aggravating the negative emotion. Furthermore, cognitive restructuring, a recommended treatment designed to increase the flexibility of the client’s thinking and gain multiple flexible perspectives, was recognized to alleviate GAD [17]. It has been suggested that vulnerability to the development of anxiety could be partially due to biased information processing, especially bias in attention to and interpretation of internal/external stimuli associated with the threat [47]. Reappraisal represents an adaptive emotion regulation strategy, which requires an intentional shift of one’s appraisal of the situation and related cognitive/emotional responses [26]. This ability to effectively alter or disengage from maladaptive thoughts and emotions can be defined as cognitive flexibility [48]. Cognitive flexibility is a key component of executive functioning, lack of which has been suggested and initially proved as a risk factor for psychological distress including depression and anxiety [27, 48–51].

**Fig. 1:**
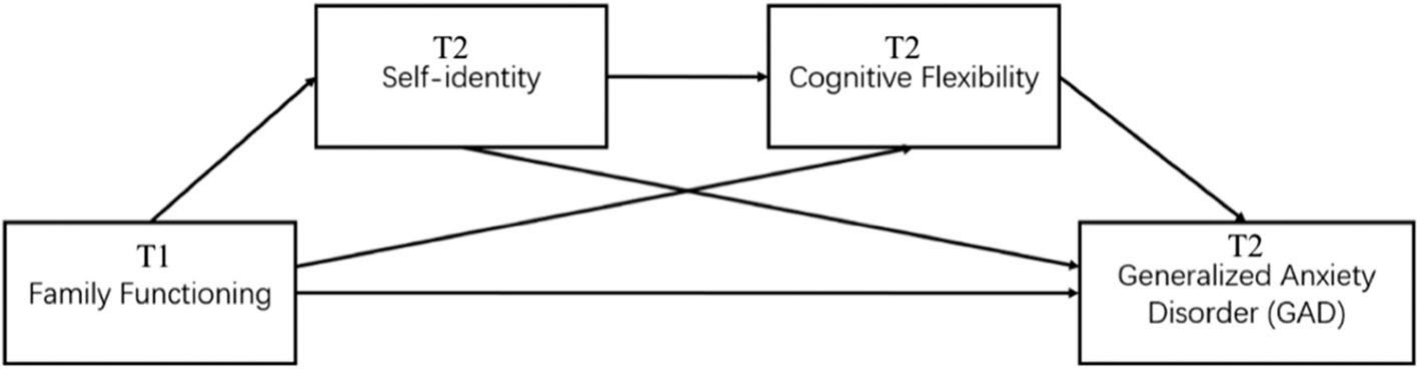
The hypothetical model

Adolescent cognitive flexibility can be influenced by negative experiences in childhood. For example, Koesten [52] found that as a fundamental factor of family functioning [53], family communication environment (i.e., family expressiveness and avoidance of conflict) positively predicted cognitive flexibility, which in turn improve adolescents’ well-being. In addition, researchers found that psychological flexibility, a similar concept to cognitive flexibility [54, 55], can mediate the relationship between chaotically-enmeshed family functioning and anxiety among young adults [56]. Based on the existing evidence, we hypothesized that poor family functioning, which could cause childhood adversity, may decrease adolescents’ cognitive flexibility and in turn increase GA symptoms. Moreover, disturbed self-identity can cause experience uncertainty and confusion, which may also exert a negative impact on cognitive flexibility [57, 58]. Given the Contrast Avoidance Model and the Avoidance Model of Worry and GAD, as well as existing studies stated above, the current study inferred self-identity and cognitive flexibility both as mediators, respectively and jointly, in the relationship between family functioning and GA. Therefore, in addition to the direct mediating effect of self-identity, we also hypothesized and examined an indirect mediating effect of self-identity on the relationship between family functioning and GA, which would be mediated by cognitive flexibility (see Fig. 1).

### The current study

The consequences of GA among adolescents were serious and the occurrence was quite common, which was worthy of our attention [6, 10]. The exploration of the mechanism of its occurrence could do benefit for the prevention and intervention of it in order to improve the mental health of the adolescents. Previous studies explored the potential precipitating factors of GA among adolescents such as family functioning, self-identity, and cognitive flexibility, but lacked a comprehensive explanation about the specific paths between the factors and roles of the factors. Moreover, most of the previous relevant studies are cross sectional. Therefore, the present study examined the longitudinal relationship between family functioning and GA in Chinese adolescents, and the mediating role of self-identity and cognitive flexibility. A two-wave design with a six-month interval was adopted. Four hypotheses were examined: (a) Family functioning (T1) would be negatively associated with GA (T2). (b) Self-identity (T2) would mediate the relationship between family functioning (T1) and GA (T2). Specifically, adolescents with poorer perceived family functioning would be more likely to generate low self-identity and thus elevated levels of GA. (c) Cognitive flexibility (T2) would mediate the relationship between family functioning (T1) and GA (T2). Specifically, adolescents with poorer perceived family functioning would show lower levels of cognitive flexibility and accordingly higher levels of GA. (d) Family functioning (T1) would indirectly predict GA (T2) through the chain mediation of self-identity (T2) and cognitive flexibility (T2) (see Fig. 1).

## Method

### Participants and procedures

According to the handbook of maximum likelihood estimation, the sample size of the current study should be 20 times the parameters used, namely at least 180 participants needed [23]. The authors contacted the headmasters of two secondary schools in Chongqing and Fujian Province, China, in order to recruit all 1613 students from the schools. After excluding those who have received intervention due to mental health problems, a total of 1548 adolescents (age from 11 to 17), from 27 randomly selected classes, completed the pencil-paper questionnaires at time (1) Of them, 1236 students (79.8%) completed the follow-up assessment at time (2) There were no significant differences in terms of the outcome variable (GA) between those who dropped out and not (*p* > 0.05). Excluding invalid answers of 13 participants, which included over 10% of missing values and the long consecutive strings of a same number, 1223 (98.95%) valid questionnaires were left (48.00% female; *M*_*age*_ = 13.14, *SD* = 1.35). Relative remaining demographic data was shown in Table 1.

**Table 1:** Demographic variables

Demographic variables	Options	*N* (/%)
One-child family	yes	568(46.4%)
	no	653(53.4%)
	missing	2(0.2%)
Residence	city	131(10.7%)
	country	954(78.0%)
	missing	138(11.3%)
Family monthly income	< 5000CNY	73(6.0%)
	5000-10000CNY	311(25.4%)
	10000-20000CNY	349(28.5%)
	> 20000CNY	207(16.9%)
	missing	283(23.2%)
Father’s education level	uneducated	4(0.3%)
	primary school	31(2.5%)
	junior high school	182(14.9%)
	senior high school	352(28.8%)
	college education	507(41.5%)
	master degree or above	93(7.6%)
	missing	54(4.4%)
Mother’s education level	uneducated	8(0.7%)
	primary school	45(3.7%)
	junior high school	209(17.1%)
	senior high school	383(31.3%)
	college education	458(37.4%)
	master degree or above	72(5.9%)
	missing	48(3.9%)

The protocol of this study was reviewed and approved by the Institutional Review Board and Ethics Committee of Faculty of Psychology, authors’ university. In the current study, written informed consent was obtained from the students. Questionnaires were mailed to teachers from each school, who were assigned to supervise students to finish them with pen and paper in class. Family functioning and GA were measured at baseline. After six months, self-identity, cognitive flexibility, and GA were measured at six-month follow-up. At last, for compensation, a debrief of the study including the information of the participant’s level of each variable was provided to each of the participant, and for those with high GA, self-help educational materials including suggestions on how to alleviate anxiety such as using progressive muscle relaxation and mindfulness skills were provided.

### Measures

#### Family adaptability and cohesion evaluation scales (FACES-II)

Family functioning was assessed using the Family Adaptability and Cohesion Evaluation Scales (FACES) [24]. In the present study, a Chinese version of the FACES validated by Fei et al. [59] was used. This 30-item scale includes two subscales, i.e., intimacy and adaptability. Items were rated on a five-point Likert scale ranging from 1 (never) to 5 (always), with higher total scores indicating healthier family functioning. The Cronbach’s alpha of this scale at T2 in the present study was 0.861. Construct validity of the scale showed excellent fit indices: Chi-square ratio (χ2 /df) = 5.696, Tucker-Lewis index (TLI) = 0.871, comparative fit index (CFI) = 0.881, and a root mean-square error of approximation (RMSEA) = 0.062, which indicated a relatively high compatibility between the model and sample data.

#### Ego identity scale (EIS)

Self-identity was measured using the Ego Identity Scale (EIS) [60]. This was a 12-item Chinese version of the original scale developed by Kato [61]. The EIS has three subscales, i.e., present devotion, past crisis, and hope for future devotion. Items were rated on a 6-point scale ranging from 1 (extremely disagree) to 6 (extremely agree). Higher total scores indicate higher developmental levels of self-identity [2]. The Cronbach’s alpha of this scale at T2 in the current study was 0.677. Construct validity of the scale showed excellent fit indices: Chi-square ratio (χ2 /df) = 4.737, Tucker-Lewis index (TLI) = 0.914, comparative fit index (CFI) = 0.952, and a root mean-square error of approximation (RMSEA) = 0.055, which indicated a relatively high compatibility between the model and sample data.

#### The cognitive flexibility inventory (CFI)

Cognitive flexibility was measured using a Chinese version of the Cognitive Flexibility Inventory (CFI) which was originally developed by Dennis and Vander Wal [62] and revised by Wang et al. [25]. The 20-item scale consists of two subscales, i.e., alternatives and control. The *alternatives* subscale was composed of 13 items which assessed the ability to perceive multiple alternative explanations for life occurrences and human behavior and the ability to generate multiple alternative solutions to difficult situations. The *control* subscale incorporates 7 items, measuring the tendency to perceive difficult situations as controllable. Higher score indicates a higher level of cognitive flexibility. Participants were invited to rate each item on a five-point scale (1 = never, 5 = always). The Cronbach’s alpha of the scale at T2 in the current study was 0.904. Construct validity of the scale showed excellent fit indices: Chi-square ratio (χ2 /df) = 6.920, Tucker-Lewis index (TLI) = 0.895, comparative fit index (CFI) = 0.915, and a root mean-square error of approximation (RMSEA) = 0.070, which indicated a relatively high compatibility between the model and sample data.

#### Generalized anxiety disorder scale-7 (GAD-7)

Generalized anxiety symptoms were measured using a Chinese version of the Generalized Anxiety Disorder Scale-7 (GAD-7) which was originally developed by Spitzer et al. [63] and revised by He et al. [64]. This 7-item scale assesses respondents’ health status during the previous two weeks. Items were rated on a three-point Likert scale ranging from 1 (never) to 3 (almost every day), with higher total scores indicating higher levels of GA symptoms. The Cronbach’s alpha of the GAD-7 in the present study was 0.892 at T1 and 0.911 at T2. Construct validity of the scale showed excellent fit indices: Chi-square ratio (χ2 /df) = 4.740 (T1) & 4.857 (T2), Tucker-Lewis index (TLI) = 0.982 (T1) & 0.984 (T2), comparative fit index (CFI) = 0.990 (T1) & 0.991 (T2), and a root mean-square error of approximation (RMSEA) = 0.055 (T1) & 0.056 (T2), which indicated a relatively high compatibility between the model and sample data.

### Data analysis

Only 1.06% of the data was missing. Expectation-maximization algorithm was used to handle missing data [23]. In view of the large sample size used in this study, we used P-P plot to test whether the relevant variables were normally distributed. The results showed that each data point in the P-P plot was akin to a straight line and basically coincided with the theoretical lines, indicating that all variables were approximately normally distributed. Means, standard deviations, Cronbach alpha coefficients, and Pearson’s correlations of the four variables assessed were computed using SPSS 26.0. Also, the current study used confirmative factor analysis to examine the model of this questionnaire. Maximum likelihood and the structural equation modeling (SEM) was conducted to test the hypothesized mediation effect for statistical significance using AMOS 21.0. Model fit was evaluated using the standards proposed by McDonald and Ho (2002)52: good model fit indices include a χ2 minimization p value above 0.05, a comparative fit index (CFI) above 0.90, a Chi-square ratio (χ2 /df) below 5.0, a Tucker-Lewis index (TLI) above 0.90, a goodness-of-fit index (GFI) above 0.90, and a root mean-square error of approximation (RMSEA) below 0.08.

## Results

### Common method bias test

Since we tested the same group of adolescents in the same measurement environment, common method bias can probably serve as a systematic error, which would have an inappropriate impact on the results. Therefore, we used Harman’s single-factor test, a most commonly employed approach, to evaluate common method bias [65]. All of the variables of interest are entered into an exploratory factor analysis. Following this, the results of the unrotated factor solution are examined to determine the number of factors that are necessary to account for the variance in the variables. In addition to that, there were 14 factors with characteristic roots over 1. The total variance extracted by one factor is 18.84% and it is less than the recommended threshold of 50%. Therefore, there’s no serious problem of common method bias in this data.

### Descriptive statistics and correlations

Means and standard deviations of studied variables as well as Pearson’s correlations among all variables are reported in Table 2. The results of the correlation analyses showed a significant correlation between family functioning, self-identity, cognitive flexibility, and GA (*p*s < 0.01).

**Table 2:** Descriptive statistic and correlations

	M	SD	1	2	3	4	5	6	7
1 Gender	587^a^	636^b^	-						
2 Age	13.14	1.35	0.06*	-					
3 T1 Family Functioning	98.47	16.70	0.01	-0.13**	-				
4 T1 GA	6.32	4.86	-0.12**	0.11**	-0.25**	-			
5 T2 Self-identity	33.31	8.43	-0.06*	-0.02	0.15**	-0.10**	-		
6 T2 Cognitive Flexibility	69.12	12.25	0.10**	-0.02	0.21**	-0.22**	0.53**	-	
7 T2 GA	6.55	5.08	-0.15**	0.01	-0.15**	0.56**	-0.13**	-0.30**	1

* Note*: ** *p* < 0.01. *p < 0.05

T1, baseline; T2, six-month follow up. *GA*, generalized anxiety. Gender was coded as 0 for female and 1 for male in the correlation analysis.

^a^number of female subjects

^b^number of male subjects

### Mediation analysis

**Fig. 2:**
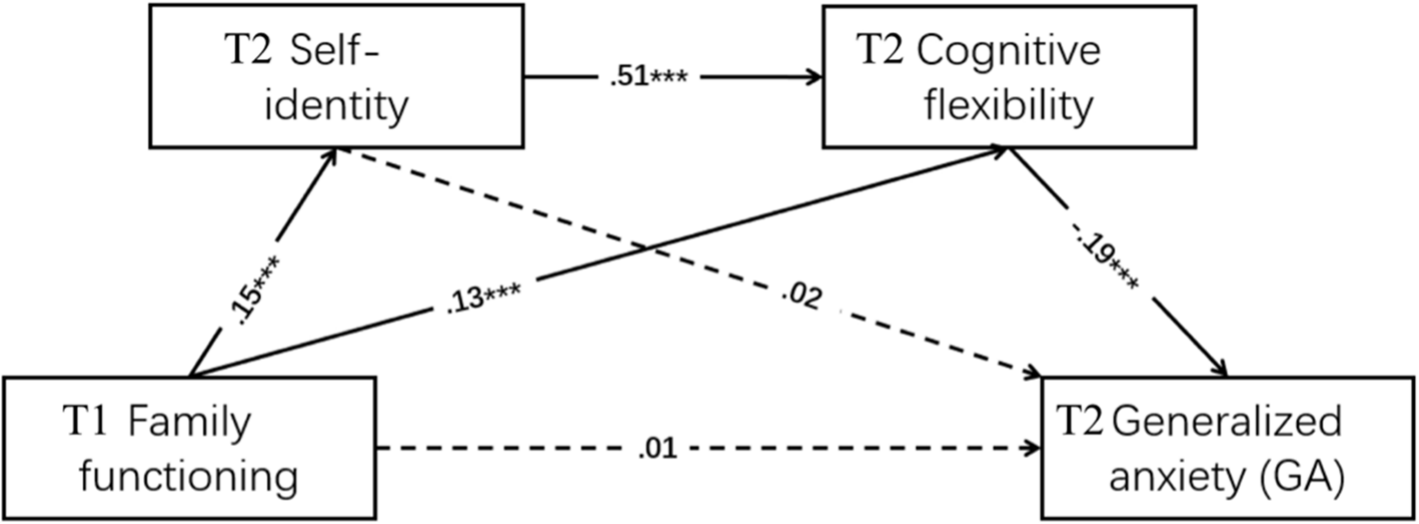
Path way of the mediation model. *Note*. ***p < 0.001

Considering the relatively high correlation between family functioning and GA at baseline, these two variables were correlated in the model. After controlling for gender, age and GA at baseline, results showed that the model had good fitness to the data, χ^2^/df = 2.922, GFI = 0.996, CFI = 0.990, TLI = 0.966, RMSEA = 0.040. The path coefficients of the model are shown in Fig. 2.

According to the output of Linear Regression, prediction of family functioning at baseline on GA at 6-month follow-up was shown as significant (*β*= -0.152, *p* < 0.001). When we applied the structural equation model to examine the mediation effects, the results showed in Table 3 indicated that the relationship between family functioning and GA was significantly mediated by cognitive flexibility (with the indirect effect=-0.008, 95% CI= -0.012 ~ -0.005) and chain-mediated by self-identity and cognitive flexibility (with the indirect effect= -0.005, 95% CI= -0.007~ -0.002). However, in this model, the relationship between family functioning and GA was not mediated by self-identity alone (with the indirect effect = 0.001, 95% CI= -0.002 ~ 0.004).

**Table 3:** The bootstrap-test for the mediation effects

Mediating Effect	Estimate	95% CI	
		Lower. 2.5%	Upper. 2.5%
Family functioning→ Self-identity→ GA	0.001	-0.002	0.005
Family functioning→ Cognitive flexibility→ GA	-0.008	-0.012	-0.005
Family functioning→ Self-identity→ Cognitive flexibility→ GA	-0.005	-0.007	-0.002

Given the significant correlations between self-identity and gender as well as between gender and GA, we conducted the model to evaluate whether gender played a moderating role in the relationship between self-identity and GA. The results revealed no moderation effect of gender (Δ*R*^2^ = 0.006, *p* > 0.05).

## Discussion

The current study examined the association between family functioning, self-identity, cognitive flexibility, and generalized anxiety (GA) using a two-wave design. Aligned with the prior findings among children in Europe and North America [9, 66, 67], the results showed that family functioning at baseline was negatively correlated with GA at 6-month follow-up among Chinese adolescents. Some important determinants of anxiety exist in the parental factors [20, 68, 69], thus the quality of family functioning accounts for the development of GA. The results also indicated that self-identity was positively correlated with family functioning and cognitive flexibility, and negatively correlated with GA, in line with previous studies [34–37, 70]. Besides, cognitive flexibility was positively correlated with family functioning at baseline and negatively correlated with perceived GA, which is also consistent with prior findings [27, 28, 48–51]. Findings in the current study indicated that among Chinese adolescents, family functioning, self-identity and cognitive flexibility can act as protective factors for the prevention for relief of GA, supporting the Contrast Avoidance model in Chinese context.

In addition, consistent with our hypothesized model, self-identity and cognitive flexibility were shown as sequential mediators between family functioning and GA. One simple interpretation of this finding is that a good level of family functioning provides adolescents with an appropriate primary environment to foster their self-identity, which is a necessary and important psychosocial development during their transition to adulthood [33]. Given that self-identity was defined as a cognitive structure originating from personality molding in adolescence [71, 72], as an ability based on cognitive structure, improved cognitive flexibility emerges due to the integrated and great self-identity building. Cognitive flexibility then enables adolescents to restrain their original internal thoughts and switch their external behavior to recognize, label, interpret, and respond to their own emotions [26, 73]. This is particularly striking in light of the fact that during adolescence cognitive flexibility preference tends to lower the risk of GA [27, 51].

Findings of the current study also revealed the mediated pathway of cognitive flexibility from family functioning to GA, which provided a new perspective for the development mechanism of adolescents’ GA. Adolescents with good family functioning can also directly struct high degree of cognitive flexibility [28, 73, 74], which provides themselves a better skill to adapt to situational conditions flexibly, thereby reducing the onset of GA.

Moreover, the mediating role of self-identity was not significant, which is not in line with our hypothesis. Specifically, the pathway from self-identity to GA was insignificant. It may be inferred that under the control of the mediating role of cognitive flexibility, the direct association between self-identity and GA acts weaker than the mediating pathway. Thus, it was not opposed to the previous research [44], but a discovery in the inter mechanism between self-identity and GA. That is, cognitive flexibility mediated the relationship between self-identity and GA. The establishment of more consummate self-identity contributes to the formation of cognitive flexibility, which makes it easier for individuals to come up with a variety of solutions in the face of crises, and they are more likely to think that crises are controllable, so that they are more adaptable to the occurrence of various negative events and reduce the occurrence of GA.

Several limitations of the current study should be mentioned. All variables were measured using self-report scales, which were subjective to the social desirability bias [75]. The common method bias test cannot guarantee that drawbacks of self-report questionnaires have no existing adverse effect on the results. Future studies should collect data using more robust approaches. Besides, there were only two time points of measurement and the time span constituted only six months in length. Two time points might not be precise and reliable enough to support mediators proceeding outcomes as well as the predictive relationship within two mediators. In addition, it should be note that alternative interpretations are possible since the longitudinal research design of the current study cannot rule out other predictive directions between variables. For instance, adolescents’ cognitive flexibility and GA may have an impact on their development of self-identity, since individuals’ self-awareness can also be influenced by emotion and cognition. Future studies are encouraged to adopt more time points and long-term follow-up assessments to improve the reliability of the study findings. Moreover, Cronbach’s alpha of the EIS (i.e., the measurement of self-identity) was relatively low in the current study, which might influence the reliability of the index of self-identity. This should be taken into consideration when interpreting the relevant results. Alternative measurements with better psychometric properties are encouraged to be used in the future research.

Findings may also offer some implications in terms of the prevention and intervention of adolescents’ GA in China. Chinese society and families have placed a lot of expectations on teenagers, in terms of academic performance and future employment. However, in the process of forming self-identity, Chinese adolescents put more emphasis on the relationship between the individual and the collective [76], and attach importance to the identification of family and social roles [77], so they produce more stress and anxiety [78, 79]. At this time, a harmonious, open and warm family, rather than an arbitrary and cold family full of contradictions and conflicts, will help teenagers reduce anxiety and grow up healthy. Simultaneously, guiding adolescents to know themselves and develop good self-identity, as well as teaching them to develop more flexible cognitive style, are helpful for them to prevent and cope with anxious emotion.

In conclusion, the present study provided preliminary evidence suggesting that adolescents with a good level of family functioning tend to show low levels of GA, with the mediating role of self-identity and cognitive flexibility. Findings in the current study expanded the exploration of mechanisms of GA in Chinese adolescents, and extended the adaptability of Contrast Avoidance Model to Chinese culture.

## Data Availability

The datasets used and/or analyzed during the current study are available from the corresponding author on reasonable request.
